# Mitochondrial Biogenesis Drives a Vicious Cycle of Metabolic Insufficiency and Mitochondrial DNA Deletion Mutation Accumulation in Aged Rat Skeletal Muscle Fibers

**DOI:** 10.1371/journal.pone.0059006

**Published:** 2013-03-13

**Authors:** Allen Herbst, Chad J. Johnson, Kayla Hynes, Debbie McKenzie, Judd M. Aiken

**Affiliations:** 1 Centre for Prions and Protein Folding Diseases, Agriculture Food and Nutritional Sciences, University of Alberta, Edmonton, Canada; 2 Department of Soil Sciences, University of Wisconsin-Madison, Madison, Wisconsin, United States of America; 3 Centre for Prions and Protein Folding Diseases, Department of Biological Sciences, University of Alberta, Edmonton, Alberta, Canada; Mayo Clinic, United States of America

## Abstract

Aged muscles possess dysfunctional fibers that contain intracellular expansions of somatically derived mitochondrial DNA deletion mutations. At high abundance, these mutations disrupt the expression of mitochondrially-encoded protein subunits of the electron transport chain resulting in aerobic respiration deficient muscle fiber segments. These fiber segments atrophy and break contributing to the loss of muscle mass and function that occurs with age. By combining micro-dissection of individual muscle fibers with microarray analysis, we observed the response induced within these abnormal muscle fibers and detected an increase in many genes affecting metabolism and metabolic regulation. The transcriptional profile and subsequent protein validation suggested that a non-compensatory program of mitochondrial biogenesis was initiated. We hypothesized that this non-adaptive program of mitochondrial biogenesis was driving mtDNA deletion mutation accumulation. We tested this hypothesis by treating aged rats with β-Guanidinopropionic acid, a compound that stimulates mitochondrial biogenesis. β-Guanidinopropionic acid treatment increased muscle mitochondrial genome copy number and resulted in a 3.7 fold increase in the abundance of electron transport chain negative muscle fiber segments. We conclude that in electron transport system abnormal muscle fiber segments, a vicious cycle of metabolic insufficiency and non-compensatory mitochondrial biogenesis drive mtDNA deletion mutation accumulation.

## Introduction

Mammalian muscle aging is characterized by the progressive non-pathological loss of muscle mass and function. Termed sarcopenia [1], this process is thought to be due to numerous diverse etiologies but primary among them are those that contribute to the individual loss of muscle fibers [2], [3]. In humans, 40% of muscle mass is lost between the ages of 20 and 80 [4], [5]. The ensuing frailty negatively impacts respiratory control, decreases mobility, predisposes towards falls and subsequent fracture, leading to the loss of independence [6]–[8].

The abundance of mitochondrial DNA (mtDNA) deletion mutations increases with advancing age in many diverse species. Although the calculated abundance of these mutations is fairly low when measured from tissue homogenates, at the cellular level, the abundance of mtDNA genomes containing deletions can be extraordinarily high, surpassing wild-type concentrations by two orders of magnitude [9]. These somatically-derived mtDNA deletion mutations accumulate clonally within cells where they ultimately disrupt expression of the mitochondrial enzymes required for electron transport and oxidative phosphorylation [9]–[13]. The mtDNA deletion mutations that accumulate within aged muscle fibers are large, typically with 5 kb or more of the 16 kb wild-type mitochondrial genome lost [14]. When present at a high intracellular abundance (>90%), the deletion mutations cause electron transport system (ETS) deficiency by disrupting the mitochondrial expression of key protein subunits of the electron transport complexes: NADH dehydrogenase, coenzymeQ:cytochrome C oxidoreductase, Cytochrome C Oxidase (COX) and ATP synthase [9]. Concomitant with the absence of enzymatic activity for these electron transport complexes is the hyperactivation of succinate dehydrogenase (SDH), the sole mitochondrial electron transport complex that is entirely encoded in the nuclear genome [15]. This dysfunction of the ETS (loss of COX and hyperactivity for SDH) is the hallmark phenotype of the mitochondrial myopathies and encephalomyopathies, a group of genetic diseases caused by mutations in the mitochondrial DNA [15]. This deficit in electron transport and oxidative phosphorylation leads to cellular atrophy, muscle fiber splitting and, ultimately, muscle fiber breakage [9], [16].

With age, the abundance of muscle fibers containing intracellular expansions of deletion mutations and associated electron transport chain dysfunction increases. In rats, by 36-months of age, *Vastus lateralis* muscle contain a 5% tissue burden of ETS abnormal fibers [17] while by 38-months of age 15% of fibers in the rat *Rectus femoris* muscle contained an ETS abnormal fiber segment [16]. In *Vastus lateralis* muscle from a 92-year old human, the tissue burden was found to be 30% at 92 years of age [18]. These measurements of ETS abnormality abundance obtained from cross-sectional studies underestimate the physiological significance of these mutations in sarcopenia, because they do not account for those fibers that have already been lost. The dynamic increase in ETS abnormal fiber segments is temporally correlated with the progressive loss of skeletal muscle fiber number that underlies the process of sarcopenia.

Muscle fiber type data also suggest a role for deletion mutations in skeletal muscle mass loss. Abnormal fibers compromised by mtDNA deletion mutation expansions predominate in muscle fibers containing type IIb myosin, fast twitch glycolytic fibers [16]. Muscles that contain the greatest proportion of type IIb fibers show the greatest muscle mass and fiber number loss with age while those muscles that conserve muscle mass and fiber number with age (type I muscles) have a low abundance of mtDNA deletion mutations-containing muscle fibers [17]. Finally, premature aging phenotypes were observed in mtDNA mutator mice that express an exonuclease defective polymerase γ, [19], [20] suggesting that mitochondrial dysfunction was involved in the physiological decline observed. Subsequent examination of tissues from these mice supported the involvement of mtDNA deletion mutations in aging phenotypes [21].

Within a muscle fiber, the accumulation of a mtDNA deletion mutation is a local phenomenon, such that a muscle fiber is genotypically and phenotypically normal along most of its length. Only a segment of the muscle fiber contains a clonal expansion of deletion-containing mtDNA molecules that compromise mitochondrial electron transport. The segmental nature of this accumulation necessitates a focal approach as homogenization would disrupt the link between genotype and phenotype, diluting the signal from the ETS abnormal region with normal tissue. To examine the cellular response to deletion mutation accumulation, we combined the selectivity of laser capture micro-dissection with the transcriptional analysis capabilities of high density oligonucleotide arrays to identify genes expressed in muscle fibers containing mtDNA deletion mutations and concomitant ETS abnormalities. We found that cells affected by intracellular expansions of deletion mutations upregulated genes involved in mitochondrial biogenesis. We hypothesize that this response results in a positive feedback loop wherein mtDNA deletion mutation accumulation results in mitochondrial dysfunction, resulting biogenesis and so enhancing deletion mutation accumulation. We tested this hypothesis by pharmacological intervention with β-Guanidinopropionic acid (β-GPA), a compound known to induce mitochondrial biogenesis [22]. We found that pharmacological intervention, in old rats, led to an increase in the incidence of ETS abnormalities. These findings suggest that pharmaceutical or environmental interventions that drive or inhibit mtDNA replication could affect mtDNA deletion mutation accumulation and ETS abnormality abundance.

## Materials and Methods

### Ethics Statement

This study was carried out in accordance with the recommendations in the NIH Guide for Care and Use of Laboratory Animals and the guidelines of the Canadian Council on Animal Care. The protocols used were approved by the Institutional Animal Care and Use Committees at the University of Wisconsin and University of Alberta.

### Animals, Tissue Preparation and Histochemistry

*Vastus lateralis* muscle was dissected from 33-month old male Fischer 344 x Brown Norway F1 hybrid rats purchased from the National Institute on Aging colony maintained by Harlan Sprague Dawley (Indianapolis, IN). The muscle was bisected at the mid-belly, embedded in optimal cutting media (Miles Inc., Elkhart, IN) and flash frozen in liquid nitrogen. Using a cryostat, 200 10-micron-thick serial transverse cryosections were cut from the frozen tissue blocks and placed on Probe-on Plus slides. At 60-micron intervals, cross-sections were stained for COX and the subsequent slide for SDH as previously described [16]. Individual muscle fibers were followed throughout the 200 microns of tissue and those that possessed regions lacking COX and were hyper-reactive for SDH activities were identified and images recording their phenotype and location obtained. All the ETS abnormal muscle fibers were exhaustively spatially identified throughout the muscle sections.

### Laser Capture Microdissection

Histological sections were dehydrated through a series of ethanol and active 1% DEPC water mixes (30%, 50%, 60%, 70%, 80%, 95%, 100% and 100%) at 4°C. Ethanol was subsequently removed with one 50% xylene:ethanol mix and two 100% xylene washes. All ETS abnormal fiber regions (840 abnormal fibers) within the tissue were micro-dissected from the sections using a PixCell II laser capture microscope (Arcturus). For each cell, the laser capture film was resected to isolate that portion of the film containing the specific cell material. The resected bits of film containing the cell were placed into RNA isolation solution. An equivalent number of ETS normal fiber sections were also micro-dissected and served as a control.

### Gene Expression Profiling

RNA isolation was performed using the MagneSil total RNA mini-isolation kit (Promega). Thirty microliters of suspended paramagnetic RNA binding particles were removed from storage buffer and added to 300 µl RNA lysis buffer to create the RNA isolation solution. The micro-dissected cells, films and paramagnetic particles were mixed and the supernatant decanted from the bound RNA/magnetic bead complexes. The magnetic beads were washed with 90% ethanol and incubated in DNAse I Digestion Solution for 15 minutes. DNAse I stop solution was added as per the manufacturer's instructions. The supernatant was removed from the RNA/magnetic bead complexes. Two washes with 90% ethanol were performed and the RNA/bead complex was allowed to dry for 10 minutes at room temperature.

The RNA was amplified by a strategy of T7 RNA polymerase-based amplification and labeling [23] using two cycles of cDNA synthesis. The purified RNA/Magnetic Bead complexes were eluted directly into the first cycle, first strand cDNA synthesis reaction prepared by using the Improm II cDNA synthesis system (Promega) according to the manufacturer's instruction using an anchored polyT-T7 promoter primer (5 µm final concentration). First cycle, second strand cDNA synthesis was performed and the entire reaction was used as template for T7 based RNA transcription using the T7 MegaScript High Yield Transcription kit (Ambion). The transcribed RNA was purified using the RNeasy mini kit (Qiagen). The nascent cRNA was subsequently subjected to a second cycle of first and second strand cDNA synthesis using random hexamer primers. The cDNA was purified using the Wizard SV Gel and PCR cleanup kit (Promega) and the sample subjected to a second cycle of T7 RNA polymerase based amplification. In this final round of T7 based RNA transcription, biotinylated 14-CTP (Invitrogen) and 16-UTP (Roche) were incorporated into the transcription/labeling reaction. The labeled amplified cRNA was purified using the RNeasy mini kit and subsequently ethanol precipitated prior to A260 quantification and fragmentation. Equal amounts of cRNA from normal and abnormal muscle fibers was chemically fragmented and hybridized to the Affymetrix rat genome 230 2.0 high-density oligonucleotide arrays according to the manufacturer's instructions (Affymetrix). NCBI GEO submission GSE35607.

### Data Analysis

The Affymetrix GCOS software package was used to identify up-regulated genes. Genes that are called present from the ETS abnormal or control chips are considered specific to the population of cells from which they came and differentially expressed unless they are detected in both ETS and control samples. Additionally, genes whose expression is greater than a ‘two fold-change’ are also considered to be differentially-regulated. Genes were annotated based upon NetAffx Annotation Release 21. Gene Ontology analysis was performed using the DAVID Bioinformatic database [24], [25].

### Immunohistochemistry

Slides containing ETS abnormal fibers were fixed in 10% buffered formalin. Antigens were retrieved by boiling in 10 mM citrate buffer, pH 6.0. Slides were blocked in TBS-T containing 5% goat serum. Primary antibodies (Table S5) were incubated in blocking solution overnight, followed by TBS-T washes. Goat anti-rabbit IgG horseradish-peroxidase conjugated antibody (1:1000) and DAB were used for detection according to the manufacturer's specification (Vector Laboratories).

### β-GPA treatment

β-guanidinopropionic acid was synthesized as described [26] from cyanamide and b-alanine, recrystallized and the synthesis confirmed by mass-spectrometry (Figure S2). Seventeen 27-month old F344/BN F1 hybrid rats were purchased from the National Institute on Aging colony. β-GPA was formulated to 1% by weight in 6% fat rodent chow (Harlan-Teklad, Madison, WI) and fed for 7 weeks *ad libitum*. Rats were housed on a 12 hour light/dark cycle. No significant difference was observed in the survival, activity, or muscle weights of rats treated with β-GPA vs controls.

### Electron Transport System abnormal muscle fiber abundance

Quadriceps muscles were removed from 28 month old β-GPA-treated and control rats and prepared for histochemistry as above. One hundred 10-micron thick serial cryosections from each animal were cut from the mid-belly of the quadriceps muscle. Serial cryosections were stained for COX, SDH, and dual stained for COX and SDH, activities along the millimeter of tissue. Dual stained sections were first stained for COX activity before being subsequently stained for SDH. Slides containing stained muscle cross sections were imaged using a Hamamatsu nanozoomer (Bridgewater, New Jersey). Screening for ETS abnormalities was performed using dual stained sections. All abnormal fiber phenotypes were confirmed by examination of the single stained COX and SDH slides. ETS abnormal muscle fiber abundance was counted from all four muscles of the quadriceps and Student's T-tests were used to determine statistical significance.

### Quantitative PCR

Quantitative PCR was performed in triplicate using the RM13645F and RM13927 primer set as described [9] on total DNA isolated from *Vastus medalis* muscles of β-GPA treated and control rats from the contralateral leg. Mitochondrial genome copy number was normalized to total DNA, β-actin gene copy number, or mitochondrial DNA standards prepared by ligating the wild-type mtDNA sequence into the pGEM T-easy vector. A standard curve was generated and the starting quantities of mitochondrial genomes were calculated. Specificity of amplification reactions was confirmed by melting point analysis and gel electrophoresis. Statistical significance was determined using Student's T-tests.

## Results

### Gene Expression Profiling of ETS abnormal muscle fibers

To identify the nuclear genome's response to the accumulation of deletion mutation-containing mtDNA genomes and the resulting ETS dysfunction, we combined histological identification of ETS abnormal fibers, in the quadriceps muscles of 36 month old rats, with laser capture microdissection and microarray analysis. Serial cross-sections of aged muscle tissue were stained for cytochrome C oxidase and succinate dehydrogenase activity, at 60 µm intervals, to identify muscle fibers containing ETS abnormal regions. Fifty-four fibers containing COX^−^/SDH^++^ regions were identified within the 2 mm length of the tissue analyzed. Eight hundred forty 10 µm thick cross-sections of ETS abnormal muscle fibers were individually collected by laser capture micro-dissection. These individual cell sections were pooled for RNA isolation, amplification and subsequent gene expression profiling. An equivalent number of ETS normal cells were collected as a control. Due to the extreme difficulty in obtaining a sufficient quantity of ETS abnormal sections of fibers and the subsequent requirement of RNA amplification, we consider the gene expression profiling to be qualitative in nature and indicative of transcripts that are present above an experimentally induced threshold determined by the RNA isolation, subsequent amplification and hybridization onto the high density gene array. Raw expression levels suggested that many transcripts were not being detected in either control or ETS abnormal sample (Figure S1). We identified 1170 unique transcripts from the ETS abnormal cell population and 750 transcripts from the control population (Tables S1 and S2 respectively). Transcripts (n = 137) detected in both samples were not considered differentially expressed.

Functional annotation of genes expressed in ETS abnormal and control skeletal muscle fibers suggested significant differences in the types of genes expressed in the two populations (Tables S3 and S4, respectively). Gene ontology terms associated with biological processes in ETS abnormal fibers were enriched for regulation and metabolic processes, consistent with the mitochondrial enzymatic dysfunction. Of the regulation GO terms, more than half were involved in the regulation of metabolism. These terms included genes for the nuclear hormone receptors estrogen related receptor alpha (esrra), retinoid X receptor alpha (rxra), neuron-derived orphan receptor (Nor1) and their coactivator ASC2/Peroxisome proliferator-activated receptor-interacting protein (NCOA6), as well as the nuclear respiratory factor 2 (gabpb2/NRF2) and the myocyte-specific enhancer factor 2a (MEF2a). All of these proteins are involved in the transcriptional control of mitochondrial gene expression, lipid oxidation and cellular metabolism [27]–[31]. These regulatory proteins positively regulate the transcription of many of the other transcripts identified in ETS abnormal fibers. GO terms associated with transcripts detected in control cells were associated with response, system and homeostatic processes, and muscle contraction.

### Immunohistochemical Validation of Gene Expression Data

**Figure 1:**
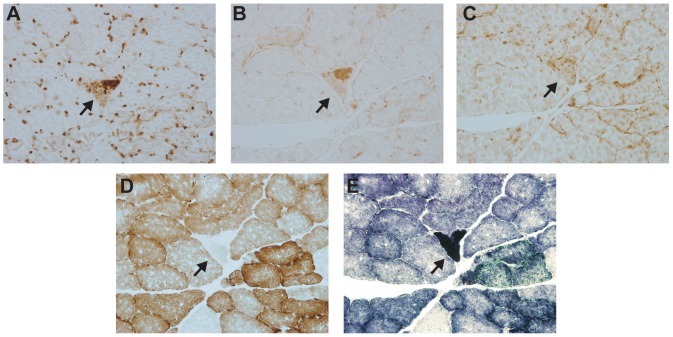
Immunohistochemical validation of genes identified in microarray experiments. A representative ETS abnormal skeletal muscle fiber is shown A. Prohibitin 2, B. Mitochondrial DNA Polymerase Gamma, C. P53 Up-regulated Mediator of Apoptosis, D. Cytochrome C Oxidase activity, E. Succinate Dehydrogenase activity.

The microarray data was confirmed immunohistochemically using antibodies against proteins whose transcripts were more abundant (Figure 1). Three proteins were selected for analysis: i) P53 up-regulated mediator of apoptosis, PUMA, ii) polymerase gamma and iii) prohibitin. We observed a focal increase in staining of ETS abnormal fibers with all three antibodies and, importantly, there was no increase in staining for these proteins in regions distant from the ETS abnormality indicating the specificity of the up-regulation to the dysfunctional segment of the fibers.

### ETS abnormal fibers are signaling to restore cellular energy homeostasis

**Figure 2:**
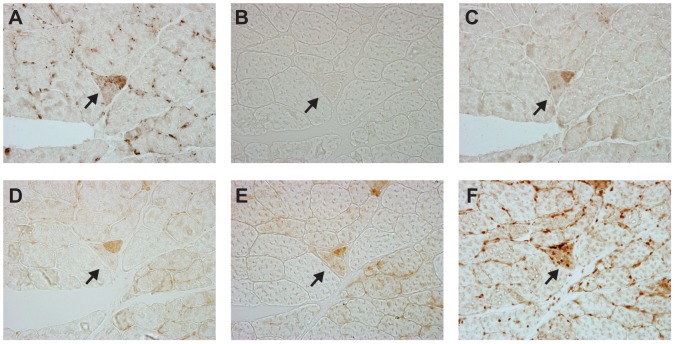
Immunohistochemical validation of signal transduction/transcriptional activation identified by gene expression profiling. Activation of AMP kinase and peroxisome proliferator activated receptor pathways in response to deletion mutation accumulation. A. CD36/Fatty acid Translocase, a pparα regulated gene, B. No Primary antibody control, C. Peroxisome proliferator-activated receptor gamma co-activator 1, D. Activated AMP Kinase, E. Inhibited Acetyl-CoA Carboxylase F. Peroxisome proliferator-activated receptor alpha.

Analysis of genes expressed in the ETS abnormal fibers suggest a pattern of dysfunctional energy homeostasis and activation of transcriptional pathways involved with metabolism, lipid oxidation and mitochondrial biogenesis. We hypothesized that the transcriptional pattern observed was due to energy deficit and dysfunctional lipid metabolism, a likely impact of loss of electron transport and oxidative phosphorylation. To test the hypothesis, we stained for activated AMP kinase and overexpression of the peroxisome proliferator activated receptor alpha (pparα) using immunohistochemistry with antibodies specific to these factors, their cofactors and their target genes. AMP kinase was phosphorylated on threonine-172, an indication of its activation, in ETS abnormal fibers. Moreover, a primary downstream target of activated AMP kinase, acetyl-coA carboxylase, was phosphorylated on serine 79, inhibiting fatty acid synthesis, an energy intensive process, consistent with an increase in AMP concentration (Figure 2).

Immunohistochemical analysis of pparα, pgc-1α (peroxisome proliferator activated receptor gamma coactivator 1 alpha) and FAT/CD36 (a pparα responsive gene), demonstrated increased protein levels for all of these factors, indicating a cellular response to the disruption of β-oxidation secondary to the loss of electron transport (Figure 2) within ETS abnormal fibers. Up-regulation of these gene products was not observed in distal ETS normal regions of the affected fibers.

### ETS abnormal fibers are induced by β-guanidinopropionic acid treatment

The localization of activated AMP kinase to skeletal muscle fiber segments with dysfunctional electron transport, second to mtDNA deletion mutation accumulation, and the up-regulation of mitochondrial DNA polymerase suggested that the cellular response to deletion mutation accumulation might positively regulate itself, driving deletion mutation accumulation. We tested the hypothesis that a program of mitochondrial biogenesis was involved in mtDNA deletion mutation accumulation by treating rats with β-guanidinopropionic acid (β-GPA), a creatine analogue that competitively inhibits creatine kinase [32], specifically interfering with the ability of skeletal muscle to regulate ATP concentration, activating AMP kinase [33] and inducing mitochondrial biogenesis [22]. β-GPA was synthesized (Figure S2) and administered perorally (1% by weight in chow) to 27-month old rats for 7 weeks.

**Figure 3:**
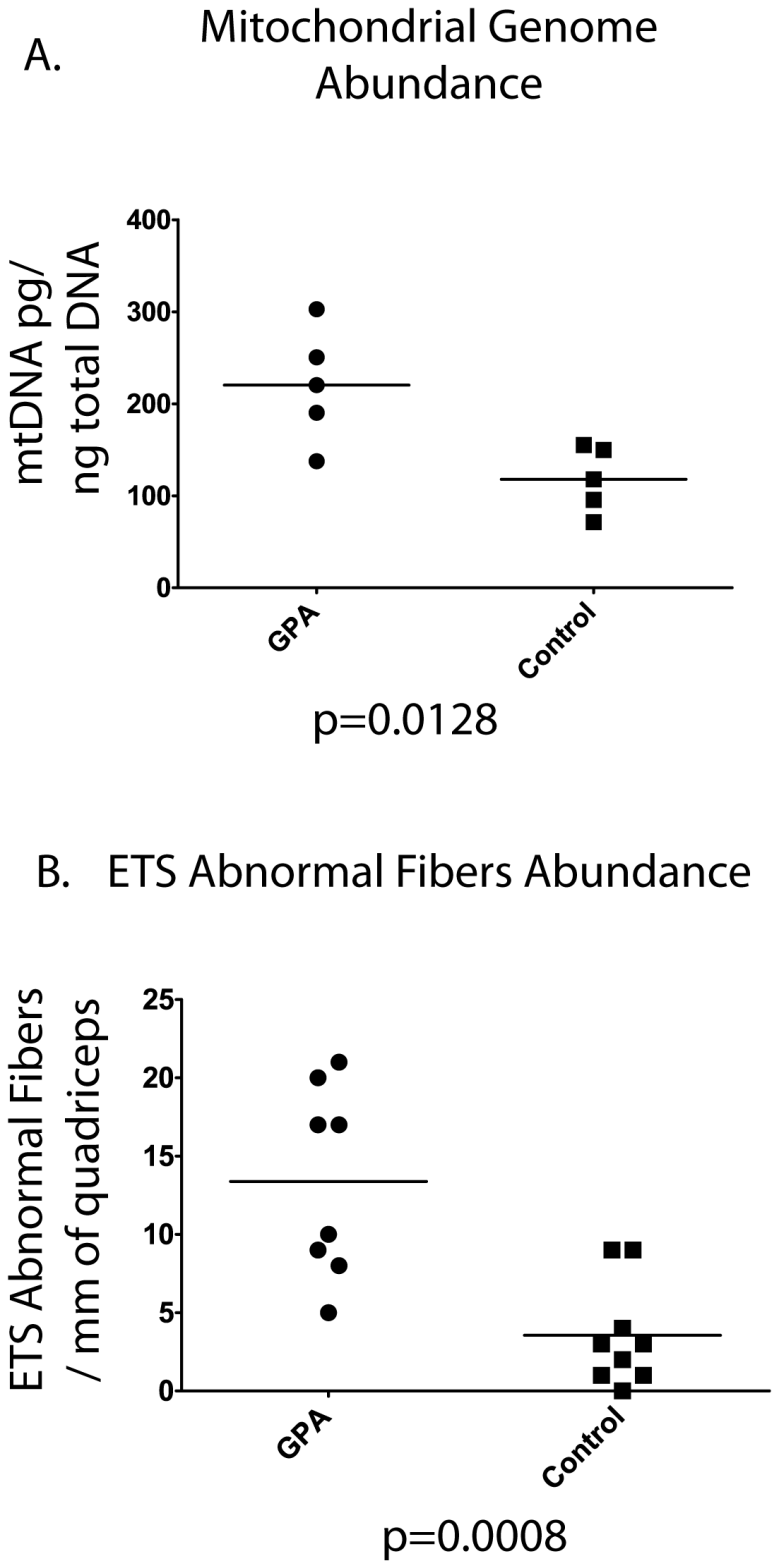
Effect of β-GPA administration on mitochondrial DNA abundance *in vivo*. A. Mitochondrial genome content of the *Vastus medialis* muscle following β-GPA treatment was measured using real-time PCR. B. Electron transport system abnormalities are more abundant in rats treated with the AMP kinase activator β-guanidinopropionic acid. Each *Vastus lateralis* skeletal muscle was stained for cytochrome C oxidase and succinate dehydrogenase every 60 microns for one millimeter. Regions of skeletal muscle fibers lacking COX activity and hyper-reactive for SDH (the ETS abnormal phenotype) were counted.

To confirm and quantify the induction of a mitochondrial biogenesis by β-GPA treatment, we used quantitative PCR to measure the total quantity of wild-type mitochondrial genomes in tissue homogenates from the *Vastus medialis* muscle. After normalizing the measurements of mtDNA obtained in the quantitative PCR reaction to account for variances in the concentration of input DNA, we detected 117 and 220 pg of mtDNA/ng of sample from control and GPA treated samples, respectively (Figure 3a). This greater than two-fold increase in the absolute number of mitochondrial genomes indicates that β-GPA treatment stimulated mitochondrial DNA replication.

To examine the effect of β-GPA treatment on the number of ETS abnormal fibers, we counted the absolute number of ETS abnormal regions within a 1-mm length of sectioned muscle (analyzing one hundred 10 µm sections) of *quadriceps* muscle from GPA-treated and control rats. We found a 3.7 fold increase in the abundance of ETS abnormal fibers in the skeletal muscles of old animals treated with GPA (P<0.0008) (Figure 3b). ETS abnormalities are first detected in muscle fibers, in the F344/BN F1 hybrid rat, between 27 and 30 months of age. In the β-GPA treated animals (28.5 months old), an average of 13.3 ETS abnormal fibers were identified while control animals had 3.5 within the millimeter of tissue examined.

## Discussion

Despite the identification of the relationship between mitochondrial DNA deletion mutation accumulation and metabolic dysfunction, the specific mechanism(s) that originate and allow clonal accumulation of mtDNA deletion mutations with aging are enigmatic. The loss of electron transport activity in muscle fiber segments harboring intracellular clonal expansions of mtDNA deletion mutations suggests that many metabolic pathways, both anabolic and catabolic, would be affected by the inability to dispose of reducing equivalents generated by respiration. Since many of the enzymes in the citric acid cycle are susceptible to product (NADH) inhibition, the electron flux would decrease, and the central hub of metabolism would be compromised. This would have a direct effect on mitochondrial ATP synthesis, and result in the requirement for the use of inefficient compensatory biochemical pathways, depleting cellular ATP concentration. We tested whether the response to electron transport dysfunction induced by the expansion of mtDNA deletion mutations was non-adaptive and consistent with the proposed role for mitochondrial deletion mutations in sarcopenia. To better understand the molecular basis of sarcopenia, we profiled (Tables S1 and S2) muscle fibers containing intracellular expansions of deletion-mutation containing mitochondrial DNA. The profile obtained suggested that AMP kinase, the ubiquitous energy sensing molecule, was activated as was nuclear hormone signaling, a response indicating a program of mitochondrial biogenesis was activated, consistent with the observed mitochondrial dysfunction in deletion mutation containing muscle fibers. Our expression profile from ETS abnormal aged muscle fibers detected common transcripts and gene products with other expression profiles from diverse models of mitochondrial disease: AMPK [34], CD36 [35], [36], Prohibitin [35], QPRT [37], pgc-1α [38] and mitochondrial Creatine Kinase [35], a protein known to form para-crystalline inclusions visible by electron microscopy in mitochondrial myopathy patients[39], [40].

We hypothesized that cells harboring clonal expansions of mitochondrial DNA deletion mutations respond to the metabolic defect caused by dysfunctional oxidative phosphorylation by up-regulating mitochondrial biogenesis, non-adaptively driving the replication of mitochondrial DNA deletion mutations. The massive accumulation of deletion-containing genomes within skeletal muscle fibers is indicative of a program of mitochondrial DNA replication allowing deletion mutations to accumulate to high levels. The coordinate up-regulation of mitochondrial DNA polymerase gamma and PEO1, the mitochondrial helicase twinkle in ETS abnormal fibers (Table S1), provides a coherent explanation for the expansion of mitochondrial genomes as over-expression of these two proteins is sufficient for mitochondrial genome proliferation *in viv*o [41]. The activation of AMP kinase suggests that accumulation of AMP initiates signaling for mitochondrial biogenesis and metabolic processes. The loss of β-oxidation would allow for the accumulation of long-chain fatty acids, potent endogenous pparα agonists. Further the expression of pgc-1α is a known inducer of mitochondrial biogenesis[42]. The cellular response to the lack of mitochondrial electron transport and oxidative phosphorylation attempts to correct the defect by up-regulating genes responsible for mitochondrial DNA replication and metabolism. This response is non-adaptive and stimulates further deletion mutation accumulation through the expression of polymerase γ and PEO1/twinkle, expanding the cellular defect.

We tested this hypothesis by initiating, pharmacologically, a program of mitochondrial biogenesis in skeletal muscle fibers from late middle-aged rats, an age when the rats typically have very low numbers of ETS abnormal fibers. ETS abnormal fibers are first observed in the VL muscle of F344/BN F1 hybrid rats at 27 months of age [43]. We used a pharmacological inhibitor , of creatine kinase, β-GPA, a muscle-specific enzyme responsible for buffering high energy phosphate during muscle contraction. Our *de novo* induction of ETS abnormal fibers is the first example of a treatment that increases the tissue burden of ETS abnormalities and provides a useful model for further studies of ETS abnormality abundance and sarcopenia. Moreover, this induction suggests that nuclear regulation of mitochondrial biogenesis expression can directly influence the accumulation of deletion mutations, evidence that deletion mutation accumulation is not merely a stochastic process.

β-GPA treatment caused a two-fold increase in wild-type mitochondrial genomes in tissue homogenates of the *Vastus medialis* muscle (Figure 3) demonstrating an induction of mitochondrial biogenesis and genome replication. This result is similar with a previously reported β-GPA treatment [33] which also increase mitochondrial genome content in muscle by 2-fold. This β-GPA induced increase in mitochondrial genomes is not due to an increased abundance of ETS abnormal fibers, or the deletion mutation expansions contained within them, as the primer sets used would not amplify deletion-containing genomes [14].

**Figure 4:**
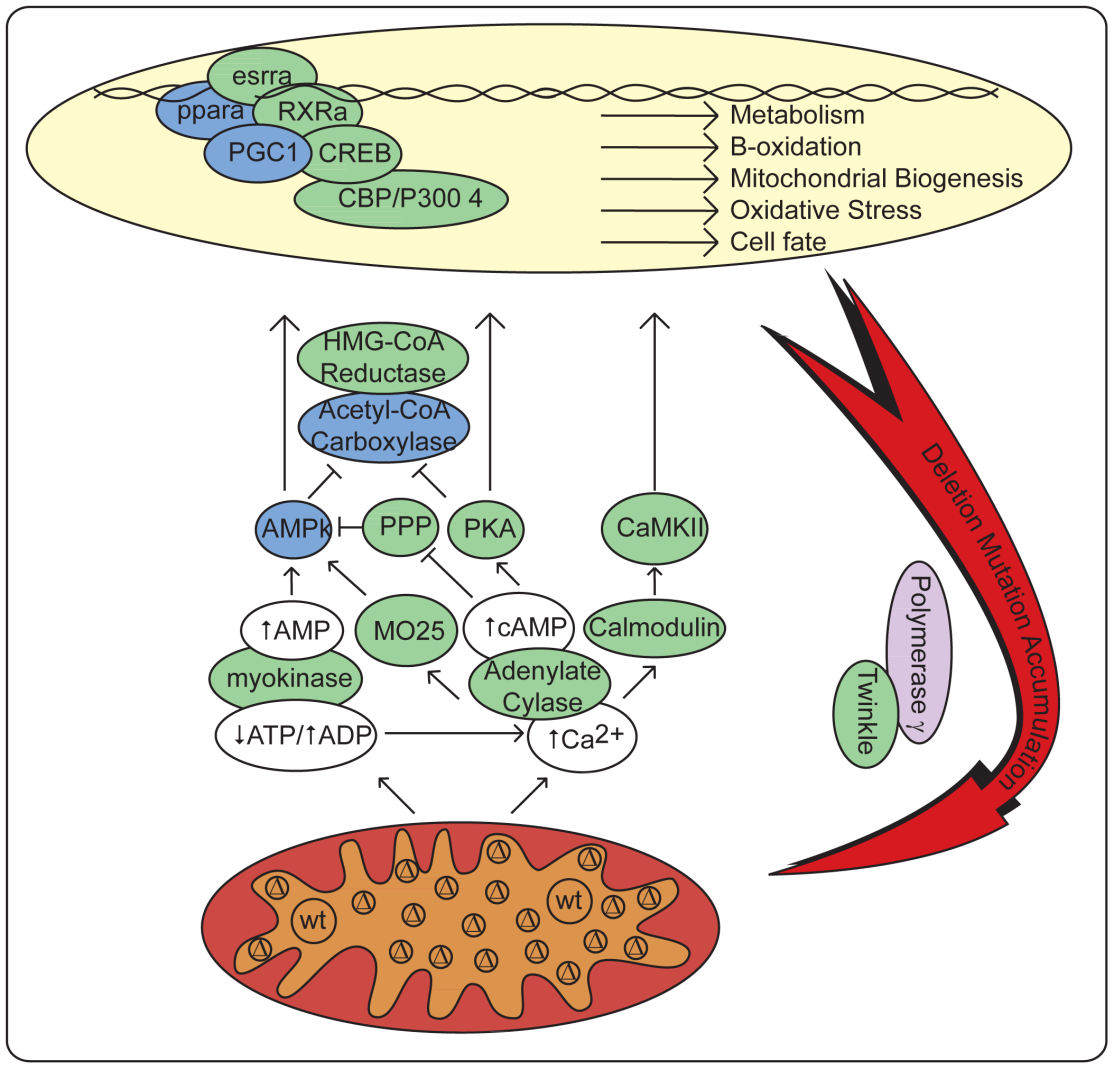
Model of positive feedback loop in ETS abnormal fibers. Signal transduction pathways detect mitochondrial dysfunction and drive transcriptional activation leading to up-regulation of mitochondrial DNA replication and subsequent deletion mutation accumulation. Genes in green were up-regulated in ETS abnormal fibers. Proteins in blue were found to be up-regulated by immunohistochemistry in ETS abnormal fibers. Proteins in purple were detected by both assays. wt: wild-type mitochondrial genomes, Δ deletion mutation containing mitochondrial genomes.

The 3.7 fold increase in the number of ETS abnormal muscle fibers in 28.5 month old, β-GPA-treated rats suggests that a program of mitochondrial biogenesis is driving the accumulation of mtDNA deletion mutations, creating a vicious cycle of mitochondrial DNA deletion mutation accumulation, metabolic dysfunction, subsequent mitochondrial biogenesis and further deletion mutation expansion (Figure 4). These data strongly suggest a critical role for mitochondrial biogenesis in age-induced deletion mutation accumulation and demonstrates the potential for perturbations to impact the abundance of mitochondrially compromised cells.

## Supporting Information

Figure S1 **Scatter plot of gene expression values.** Genes detected in ETS abnormal fibers are not found in control fibers and vice versa, necessitating a qualitative approach to analysis.
(DOCX)

Figure S2 **Confirmation of the synthesis of β-guanidinopropionic from β-alanine and cyanamide.** The electrospray ionization, time-of-flight mass spectrum shows β-GPA and it's zwitterionic multi-mers in various hydration states.
(DOCX)

Table S1 **Genes detected in ETS abnormal Fibers.**
(XLSX)

Table S2 **Genes detected in Control Fibers.**
(XLSX)

Table S3 **Gene Ontology terms enriched in ETS abnormal Fibers.**
(XLSX)

Table S4 **Gene Ontology terms enriched in Control Fibers.**
(XLSX)

Table S5 **Antibodies for immunohistochemistry, dilution used and source.**
(DOCX)
