# Supplementary material for: Mitochondrial Biogenesis Drives a Vicious Cycle of Metabolic Insufficiency and Mitochondrial DNA Deletion Mutation Accumulation in Aged Rat Skeletal Muscle Fibers
Source: PLoS One. 2013 Mar 13;8(3):e59006. doi: 10.1371/journal.pone.0059006 (PMC3596334; doi:10.1371/journal.pone.0059006)
Supplement: Figure S1 — Scatter plot of gene expression values. Genes detected in ETS abnormal fibers are not found in control fibers and vice versa, necessitating a qualitative approach to analysis. (DOCX) [file pone.0059006.s001.docx]

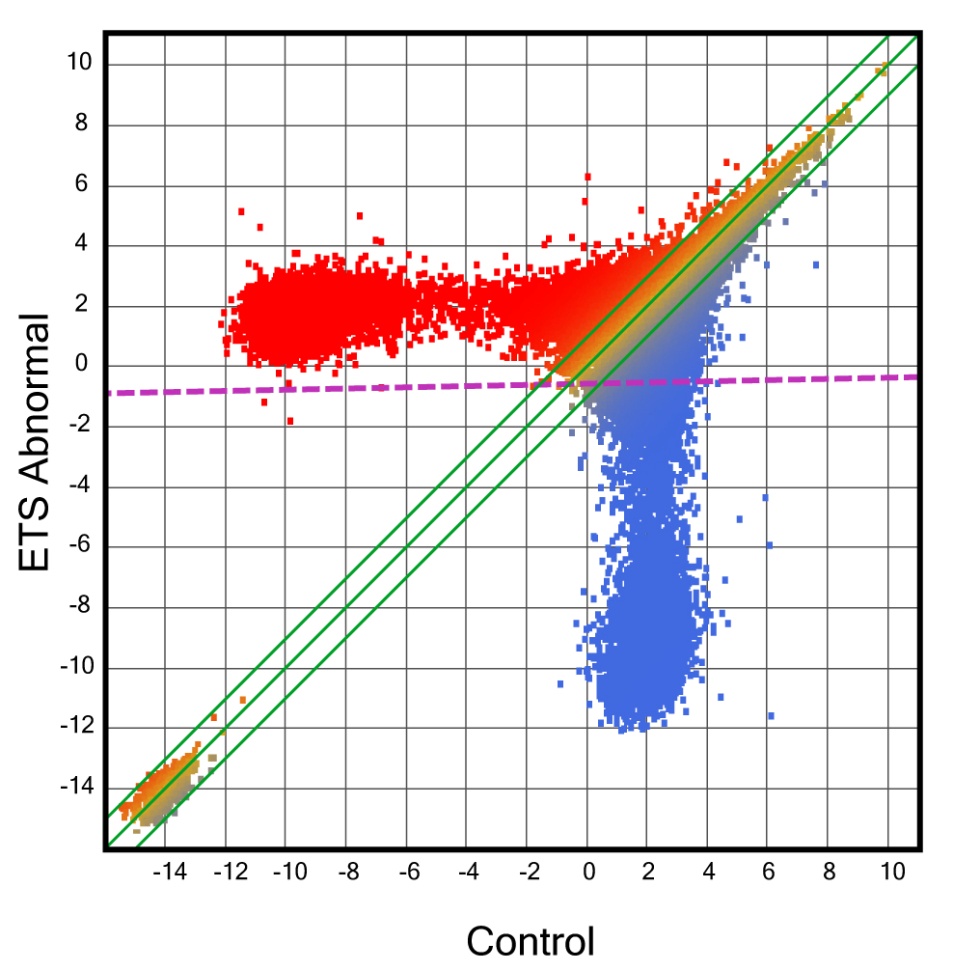


Figure S1. Scatter plot of gene expression values. Genes detected in ETS abnormal fibers are not found in control fibers and vice versa, necessitating a qualitative approach to analysis.
