# Supplementary material for: Mitochondrial Biogenesis Drives a Vicious Cycle of Metabolic Insufficiency and Mitochondrial DNA Deletion Mutation Accumulation in Aged Rat Skeletal Muscle Fibers
Source: PLoS One. 2013 Mar 13;8(3):e59006. doi: 10.1371/journal.pone.0059006 (PMC3596334; doi:10.1371/journal.pone.0059006)
Supplement: Figure S2 — Confirmation of the synthesis of β-guanidinopropionic from β-alanine and cyanamide. The electrospray ionization, time-of-flight mass spectrum shows β-GPA and it's zwitterionic multi-mers in various hydration states. (DOCX) [file pone.0059006.s002.docx]

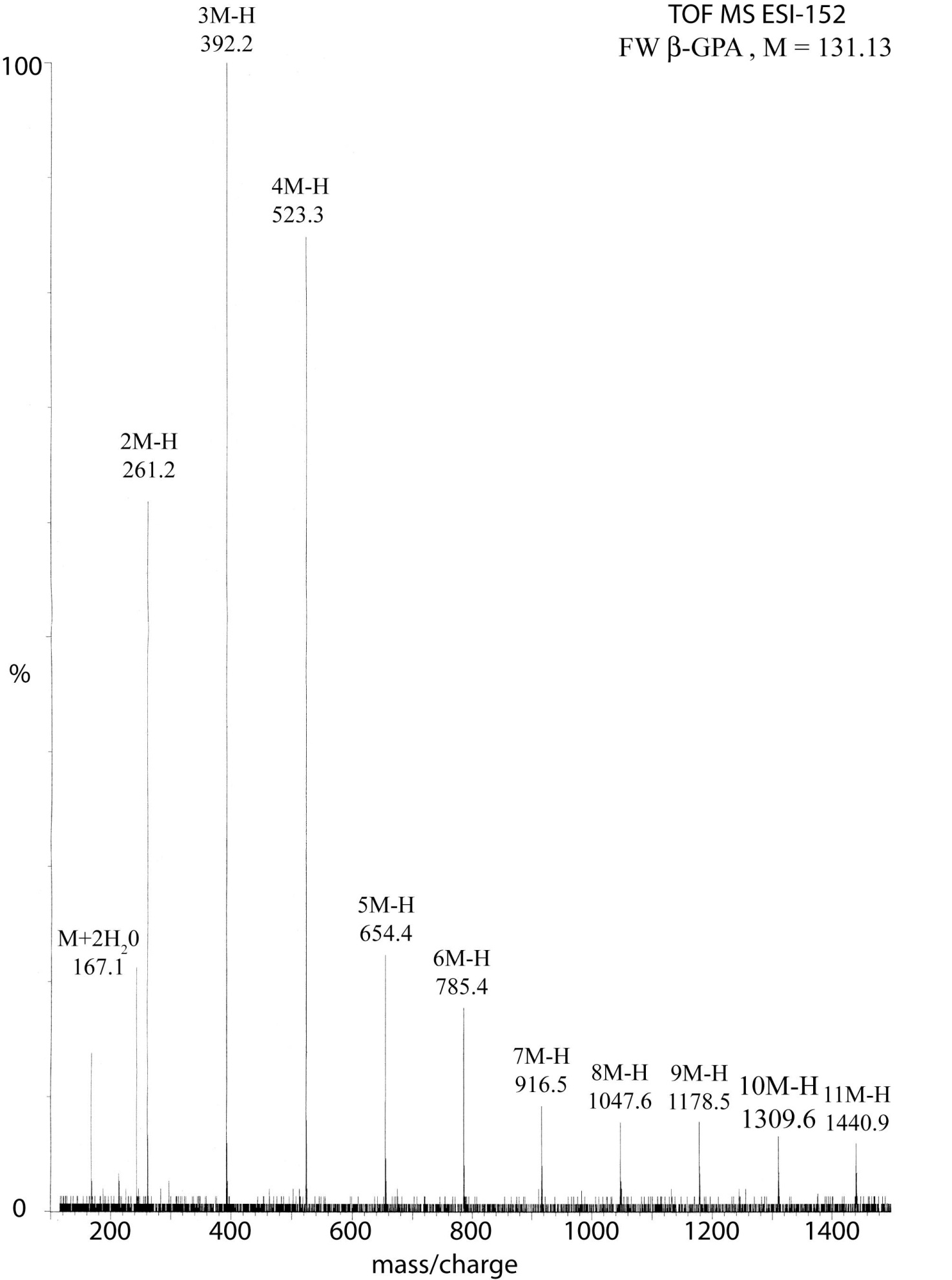


Figure S2. Confirmation of the synthesis of β-guanidinopropionic from β-alanine and cyanamide. The electrospray ionization, time-of-flight mass spectrum shows β-GPA and it's zwitterionic multimers in various hydration states.
