# Supplementary material for: Mitochondrial Biogenesis Drives a Vicious Cycle of Metabolic Insufficiency and Mitochondrial DNA Deletion Mutation Accumulation in Aged Rat Skeletal Muscle Fibers
Source: PLoS One. 2013 Mar 13;8(3):e59006. doi: 10.1371/journal.pone.0059006 (PMC3596334; doi:10.1371/journal.pone.0059006)
Supplement: Table S5 — Antibodies for immunohistochemistry, dilution used and source. (DOCX) [file pone.0059006.s007.docx]

Table S5. Antibodies for immunohistochemistry, dilution used and source.

| Antigen | Dilution | Supplier |
| --- | --- | --- |
| PUMA | 1:200 | Cell signaling Technologies |
| Phospho-Acetyl-CoA Carboxylase (ser79) | 1:200 | Cell signaling Technologies |
| Phospho-AMPKα (Thr172) | 1:20 | Cell signaling Technologies |
| PPARα | 1:100 | Cayman Chemical |
| PGC-1 | 1:100 | Cayman Chemical |
| Prohibitin 2/Rea | 1:100 | Bethyl Laboratories |
| FAT/CD36 | 1:200 | Lifespan Biosciences |
| DNA polymerase γ | 1:200 | Abcam Inc. |
